# Assessment of Frequency, Patterns, and Causes of Blunt Abdominal Trauma in a North Indian Cohort: An Autopsy-Based Study

**DOI:** 10.7759/cureus.44856

**Published:** 2023-09-07

**Authors:** Saroj Kumar Ranjan, Ritesh Kumar Singh, Sanjeev Kumar, Pinki Kumari

**Affiliations:** 1 Forensic Medicine and Toxicology, Indira Gandhi Institute of Medical Sciences (IGIMS), Patna, IND; 2 Forensic Medicine and Toxicology, Nalanda Medical College and Hospital (NMCH), Patna, IND

**Keywords:** abdomen, observational study, road traffic accidents, trauma, blunt abdominal injuries

## Abstract

Introduction

The abdomen is one of the most frequently damaged areas in trauma patients and is commonly encountered in road traffic accidents (RTAs). The present study evaluates the frequency, etiology, causation, and form of injury in blunt abdominal trauma (BAT) cases who had autopsies.

Method

An autopsy-based observational prospective study was conducted at the Department of Forensic Medicine and Toxicology, Nalanda Medical College and Hospital Patna, India, during the period from October 2018 to September 2020, which included a total of 940 autopsy cases showing blunt abdominal injuries. A predesigned proforma for the postmortem evaluation of BAT victims was used to collect the required information on the cases. Descriptive statistics were performed, and the data were presented as frequency (%) and mean±SD. Chi-square tests were performed to compare categorical variables between groups.

Results

BAT accounted for 120 (12.76%) cases of all autopsies performed. The majority of victims were male (92.48%). Victims aged 21-30 years (31, 25.83%) were the ones most usually engaged in BAT cases. Among the mechanisms accountable for BAT, RTAs were the most common (99, 82.50%), followed by assault (16, 13.30%). In most of the cases, the liver was injured (107, 89.16%), followed by the spleen (60, 50.00%) and kidney (24, 20.00%). The majority of blunt abdominal injury-related deaths were accidental (100, 83.33%), followed by homicidal (15, 12.5%) and suicidal cases (5, 4.17%). Hemorrhage and neurogenic shock were the most prevalent causes of mortality, particularly if the individual died within a few hours.

Conclusion

RTAs are the most frequent cause of BAT in autopsy cases, and the liver is the most affected organ. The majority of deaths occur within the first 24 hours of injury. Since blunt abdominal injuries have the propensity to increase morbidity and mortality, appropriate emphasis on their precise diagnosis and satisfactory therapy is mandated.

## Introduction

Trauma continues to be the leading cause of mortality in people below 40 years of age, and it is a serious issue worldwide [1]. The abdomen is the most commonly wounded site, and surgical intervention is necessary in about one-fourth of noted incidences [2]. Blunt abdominal trauma (BAT) and penetrating abdominal trauma (PAT) are the two most important types of abdominal trauma [3]. PAT is quickly and consistently identified, whereas BAT is frequently overlooked as medical indications for blunt abdominal injuries are fewer. PAT is frequently classified into stab wounds and gunshot wounds. A previous study on significant BAT highlighted that primarily extra-abdominal injuries (77.6%) attributed more to deaths as compared to intra-abdominal injuries (8.4%) [4]. According to Costa et al., the most prevalent causes of BAT include motor vehicle collisions (MVCs), falls from great heights, and assaults [5]. Gunshots, stabbing, and other things that invade the peritoneal cavity are the most common causes of penetrating trauma.

Blunt trauma in the abdomen is generally overlooked during the initial assessment. This postponement of diagnosis and insufficient therapy can be lethal in the long run. It is critical that clinical examination be supplemented with radiological imaging techniques to make a diagnosis and discern visceral abdominal and chest injuries. Solid visceral injuries encompass the spleen, liver, and kidney and are characterized by symptoms of shock, whereas enteric injuries are characterized by peritonitis and sepsis [6,7].

Statistics on the origin and prognosis of abdominal injuries in our region are few. Therefore, the major goal of our study was to evaluate the frequency, etiology, causation, and form of injury in autopsy cases with BAT.

## Materials and methods

Selection of cases

This autopsy-based observational prospective study was conducted at the mortuary of the Department of Forensic Medicine and Toxicology, Nalanda Medical College and Hospital Patna, India, from October 2018 to September 2020. A total of 940 medicolegal autopsy cases showing blunt abdominal injuries were enrolled in the study.

Inclusion criteria

All the autopsy cases in which there was a known cause of BAT were included in the study. Also, all the BAT cases (with or without associated body injuries) who were hospitalized post-accident but succumbed to the injuries were included in the study.

Exclusion criteria

PAT cases and cases in which the clear cause or nature of trauma was unknown or in which the bodies were decomposed were not included in the study. All the survivors were excluded from the study. 

Ethical approval

Ethical approval was obtained from the Institutional Review Board of Nalanda Medical College and Hospital, Patna, India (NMCH/2018/FM-098).

Data collection

All the relevant information was collected in a predesigned proforma for the postmortem evaluation of BAT victims. Information such as history, causal factors, external examination, internal examination, associated injuries, and cause of death were entered into a Microsoft Excel sheet.

Statistical analysis

Data entered in Microsoft Excel sheets were subjected to statistical analysis. IBM SPSS Statistics for Windows, Version 21.0 (Released 2012; IBM Corp., Armonk, New York, United States) was used to perform the statistical analysis. Descriptive statistics were performed, and the results were presented as frequency (%). Chi-square tests were performed to compare categorical variables between groups, and a p-value of less than 0.05 was considered to be statistically significant.

## Results

Incidence of BAT in autopsy cases

Out of the 940 autopsies, 120 cases (12.76%) involved BAT (Table 1).

**Table 1:** Incidence of BAT in autopsy cases. BAT: blunt abdominal trauma.

Type of case	Cases	
	N	%
BAT	120	12.77
Others	820	87.23
Total	940	100

Incidence of causative factors of BAT in autopsy cases

The most common causative factor associated with BAT in the autopsy cases studied was road traffic accidents (RTAs), comprising 99 cases (82.50%) of the total 120 cases, followed by assault, comprising 16 cases (13.30%) of the total cases (Table 2).

**Table 2:** Causative factors of BAT in the autopsy cases. BAT: blunt abdominal trauma; RTA: road traffic accident; FH: fall from height; UF: unconscious fall; COR/DHO: collapse of roof/drop of heavy object.

Mode of injury	N	%
Assault	16	13.30
RTA	99	82.50
FH	2	01.70
UF	0	0.00
COR/DHO	0	0.00
Train accident	3	2.50
Others	0	0.00
Total	120	100

Age and sex incidence

Among the 120 autopsy cases who had BAT, 112 (93.33%) were male and 8 (6.67%) were female. Most of the BAT cases were observed in the 21- to 30-year age group, with 31 cases (25.83%) out of the total 120 cases. The second-most represented age group was 11-20 years, with 29 cases (24.17%) out of the total cases. The least represented age groups were 0-10 years and above 70 years, with only one case in each group (0.83%). There was no significant difference in the age distribution among males and females (p=0.08) (Table 3).

**Table 3:** Age- and sex-wise distribution of cases. BAT: blunt abdominal trauma.

Age (in years)	Male		Female		Total		Chi-square p-value
	N	%	N	%	N	%	0.08
0-10	1	0.83	1	0.83	2	1.67	
11-20	29	24.17	0	0.00	29	24.17	
21-30	28	23.33	3	2.50	31	25.83	
31-40	24	20.00	0	0.00	24	20.00	
41-50	21	17.50	3	2.50	24	20.00	
51-60	6	5.00	1	0.83	7	5.83	
61-70	2	1.67	0	0.00	2	1.67	
71-80	1	0.83	0	0.00	1	0.83	
Total	112	93.33	8	06.67	120	100	

Abdominal viscera involved

The incidence of various organs involved in BAT in the autopsy cases is shown in Table 4. The highest incidence was observed for the liver (107, 89.16%), followed by the spleen (60, 50.00%) and kidney (24, 20.00%) (Table 4).

**Table 4:** Abdominal viscera involved in autopsy cases with BAT. BAT: blunt abdominal trauma.

Viscera involved	N	%
Liver	107	89.16
Spleen	60	50.00
Intestine	17	14.16
Stomach	7	5.83
Omentum	5	4.16
Mesentery	10	8.33
Kidney	24	20.00
Adrenals	4	3.33
Bladder	5	4.16
Pancreas	4	3.33
Uterus	2	0.66
Gall bladder	0	0.00

Mode of injury to abdominal organ

The relation between abdominal organ injury and its mode of injury is shown in Table 5. RTA was the most prevalent mode of injury to all the visceral organs except for the omentum where the most prevalent mode of injury was assault (Table 5).

**Table 5:** Mode of injury in autopsy cases with BAT. BAT: blunt abdominal trauma; RTA: road traffic accident; FH: fall from height; UF: unconscious fall; COR/DHO: collapse of roof/drop of heavy object.

Viscera involved	Frequency of mode of injury						
	Assault	RTA	FH	UF	COR/DHO	Train accident	Others
Liver	12	90	2	0	0	3	0
Spleen	6	50	1	0	0	3	0
Intestine	5	8	0	0	0	1	0
Stomach	3	4	0	0	0	0	0
Omentum	4	1	0	0	0	0	0
Mesentery	5	5	0	0	0	0	0
Kidney	2	14	0	0	0	1	0
Adrenals	0	4	0	0	0	0	0
Bladder	0	4	0	0	0	0	0
Pancreas	1	3	0	0	0	0	0
Uterus	0	2	0	0	0	0	0
Gall bladder	0	0	0	0	0	0	0

Frequency of multiple abdominal organs involved

The majority of the autopsy cases with BAT involved two organs (45, 37.50%), liver and spleen, followed by the involvement of one organ (43, 35.83%) (Table 6).

**Table 6:** Frequency of multiple abdominal organs involved in the autopsy cases with BAT. BAT: blunt abdominal trauma.

Number of organs involved	N	%
One organ	43	35.83
Two organs	45	37.50
Three organs	20	16.67
Four organs	7	5.83
Five or more organs	5	4.17
Total cases	120	100

Associated injuries

Six cases (5%) out of the total autopsy cases with BAT showed only abdominal injuries without any other associated injuries. The most frequent associated injuries involved the chest (7, 89.16%) and limbs (83, 69.16%) (Table 7).

**Table 7:** Other body parts injured in autopsy cases with BAT. BAT: blunt abdominal trauma.

Part of body injured	N	%
Head	73	60.83
Chest	107	89.16
Spine	0	0.00
Limbs	83	69.16
Pelvis	9	7.50
Abdomen alone	6	5.00

Period of survival

Out of the 120 autopsy cases with BAT, 58 (48.33%) were either spot dead, which means they passed away immediately after suffering an injury; brought dead, which means they died later and were pronounced "Brought dead" when they arrived at the hospital's casualty, or those who succumbed to their injuries less than two hours after suffering the injury (Table 8).

**Table 8:** Period of survival in autopsy cases with BAT. BAT: blunt abdominal trauma.

Survival period	No. of cases	Percentage
<2 hours	58	48.33
2-6 hours	0	0.00
6-12 hours	0	0.00
12-24 hours	3	2.50
1-3 days	3	2.50
3-7 days	0	0.00
1-2 weeks	0	0.00
>2 weeks	0	0.00
Unknown	56	46.67

Cause of death

Neurogenic shock, hemorrhagic shock, and septicemia, either occurring alone or in combination with one another, were the most observed causes of death in autopsy cases with BAT (Table 9).

**Table 9:** Cause of death in autopsy cases with BAT. BAT: blunt abdominal trauma.

Cause of death	No. of cases	Percentage
Hemorrhage	69	57.50
Septicemia	6	5.00
Neurogenic shock	0	0.00
Hemorrhage + neurogenic shock	45	37.50

Nature of death

Out of the 120 autopsy cases with BAT, accidental deaths comprised 100 (83.33%) cases, followed by homicidal cases (15, 12.5%) and suicidal cases (5, 4.17%) (Table 10).

**Table 10:** Nature of death in autopsy cases with BAT. BAT: blunt abdominal trauma.

Nature of death	No. of cases	Percentage
Accidental	100	83.33
Suicidal	5	4.17
Homicidal	15	12.50
Total	120	100

## Discussion

Trauma is the leading cause of illness and death in young people all across the world. It is a reported fact that RTA will be a prevalent cause of disability worldwide and the second-most prevalent factor in developing nations [8].

The predominance of our autopsy cases belonged to the young productive age group of 21-30 years, which was noted to be consistent with earlier research conducted elsewhere [9,10]. We observed a male preponderance in our study, similar to the findings reported by Fleming et al. [11].

Consistent with the findings of other studies, we also observed RTA to occur with maximum frequency among the factors responsible for BAT in the autopsy cases studied [8,10,12]. Such occurrences may be attributed to increased motorization or inadequate road facilities in the studied area. These insights are critical for developing preventative methods to minimize RTAs and the consequent occurrence of trauma. Factors that have contributed to a rise in the frequency of fatal accidents in and around Ranchi include the occurrence of cars on the road, overcrowding, and, to a significant degree, a dearth of traffic sense, particularly among bus and truck drivers. Pedestrians crossing roadways at random, particularly in congested areas, have also contributed to a rise in the death rate. Assaults, train accidents, and falls from great heights were the next most common causes of BAT observed in the study. 

RTA inflicts mechanical trauma, which leads to illness, disability, and even death. The death rate in RTAs in India is one of the highest worldwide, being more than 20 times higher than in developed countries [13]. The incidence, however, is substantially greater than that reported by Wong et al. in Singapore [14]. The fact that there were fewer occurrences in their survey might be ascribed to Singaporeans' exceptional awareness and observance of traffic laws. The trends of injuries in RTA vary greatly between developed and developing countries like India, as well as across cities within India.

The most likely consequence of abdominal trauma is blunt liver damage, which is linked with a high death rate [15]. The main causative factor for these injuries was RTAs. According to one research, the spleen was the most commonly wounded organ, as opposed to the liver, which has been found in other series [10,16,17]. However, in the present study, we observed the highest incidence for the liver (189.16%), followed by the spleen (50.00%%) in the autopsy cases with BAT. 

Blunt abdominal injuries were most commonly associated with thoracic and head injuries in the autopsy cases with BAT in the present study. The findings supported previous findings where chest injuries were the most prevalent documented simultaneous extra-abdominal injuries, followed by extremities and head injuries [18]. Furthermore, Mohamed et al. from Saudi Arabia observed that the most often linked extra-abdominal injuries were chest and head traumas [19].

Hemorrhagic shock in combination with neurogenic shock accounted for the majority of deaths in the autopsy cases with BAT in the present study. Another study found that a high prevalence of brain damage combined with hemorrhagic shock (60%) was the cause of mortality in cases of BAT [20]. Another study found that brain damage (47% of cases) was the leading cause of mortality, followed by visceral shock (42%) [21].

In the current study, 48.33% of patients were spot dead, brought dead, or died from their injuries within two hours of the occurrence. The majority of victims died within the first 24 hours after being injured. These observations are well backed by the previous studies that reported more than 90% mortality within the initial 24 hours of retaining BAT; other studies also reported more deaths occurring within the initial 24 hours of sustaining BAT [21-23]. More than 40% of patients survived in the first 24 hours. This stresses the fact that these patients require immediate emergency medical attention as well as a speedy transfer from the scene of the occurrence to the hospital.

According to another study, mortality occurred in 80% of instances within two days after incurring trauma [20]. Subedi et al. presented comparable results where it was reported that children and old individuals, as well as victims with connected injuries to the chest, had much longer life periods than victims with other injuries to the head, who had significantly shorter survival periods [24]. These differences in survival time with age and concomitant injuries can be extremely useful in the management of BAT situations.

Limitations

As the present study included only autopsy cases, it does not give an idea about the incidence of death. To understand the outcomes of BAT, both survivors and non-survivors should be included in the study. Therefore, further large-scale studies including both survivors and non-survivors should be conducted to comprehensively evaluate the clinical outcomes of the BAT cases.

## Conclusions

RTAs are the most frequent cause of BAT in autopsy cases. The liver is the most affected organ in these cases. The majority of the deaths occur within the first 24 hours of injury. Since traumatic abdominal injuries have the possibility of raising morbidity and mortality, appropriate emphasis on their precise diagnosis and satisfactory therapy is crucial. The survival duration can be quite useful in determining the prognosis of episodes of blunt abdominal injuries. Until demonstrated differently, all patients with head injuries hospitalized with unconsciousness and suffering shock soon after must be presumed to have intra-abdominal damage. 
